# High-Grade Glioma Recurrence Is Delayed in Hispanic Patients despite Severe Social Vulnerability: A Retrospective Cohort Study

**DOI:** 10.3390/cancers16081579

**Published:** 2024-04-20

**Authors:** Joshua A. Reynolds, Isabella L. Pecorari, Alexander Ledet, Vijay Agarwal

**Affiliations:** Department of Neurological Surgery, Albert Einstein College of Medicine/Montefiore Medical Center, Bronx, NY 10461, USA; isabella.pecorari@einsteinmed.edu (I.L.P.); alexander.ledet@einsteinmed.edu (A.L.)

**Keywords:** high-grade glioma, glioblastoma, ethnicity, time to recurrence, social vulnerability

## Abstract

**Simple Summary:**

Though diagnosed earlier, Hispanic patients with high-grade glioma experience longer survival intervals. As environmental or biologic factors are thought to influence prognosis, the aim of our retrospective study was to determine if this improvement in disease course still occurs in a socioeconomically disadvantaged sample of Hispanic patients (an environmental condition pre-disposing patients to poor oncologic outcomes). We found that despite significantly higher social vulnerability, Hispanic patients experienced an additional 14 months before the first tumor recurrence compared to non-Hispanic patients. Specifically, in those with aggressive glioblastomas, Hispanic ethnicity independently predicted an additional 8.5 months before recurrence in a multivariate analysis. These findings emphasize the need for basic science investigations into the biologic mechanisms potentially explaining Hispanic ethnicity’s influence on high-grade glioma outcomes.

**Abstract:**

High-grade gliomas (HGGs; WHO grade III or IV) are the most common and lethal brain malignancy. Patients of Hispanic ethnicity are diagnosed with HGGs earlier than non-Hispanic patients, but they exhibit improved HGG survival following diagnosis. Either environmental or biological factors could explain this survival benefit. We aimed to determine if post-diagnosis advantages would still be present in Hispanic patients with high social vulnerability, an environmental condition predisposing patients to poor oncologic outcomes. HGG outcomes were retrospectively assessed in a cohort of 22 Hispanic patients and 33 non-Hispanic patients treated for HGGs from 2015 to 2020 at a single institution that serves a highly vulnerable region. Compared to non-Hispanic patients, Hispanic patients demonstrated higher social vulnerability index scores (96.8 + 0.7 vs. 76.3 + 4.6; *** *p* = 0.0002) and a 14-month longer interval between diagnosis and recurrence (19.7 + 5.9 (n = 13) vs. 5.5 + 0.6 months (n = 19); ** *p* = 0.001). In only those patients with more aggressive IDH-1 wildtype tumors (glioblastoma), Hispanic ethnicity still related to a longer time before recurrence (15.8 + 5.9 months (n = 9); 5.5 + 0.6 months (n = 18); * *p* = 0.034), and in a multivariate analysis, Hispanic ethnicity predicted time-to-recurrence (* *p* = 0.027) independent of patient age, functional status, MGMT gene methylation, or treatments received. Therefore, environmental factors, specifically social vulnerability, did not obscure the post-diagnosis benefits associated with Hispanic ethnicity. In future experiments, basic studies should be prioritized which investigate the cellular or genetic mechanisms underlying this ethnicity effect on HGG progression in the hopes of improving care for these devastating malignancies.

## 1. Introduction

Gliomas are the most common type of malignant intracranial tumor, constituting 80.7% of these pathologies [1,2]. Large population-based studies have estimated a glioma incidence around 6 cases per 100,000 people per year in the United States [3,4,5]. Most gliomas are diagnosed in patients between the ages of 40 and 70 [6], and patients are predominately male [7,8]. Occurring through oncogenic genetic changes in glial cell types, gliomas can vary in malignant potential at presentation [9,10]. Those glia-derived malignancies with a World Health Organization (WHO) grade of III or IV are classified as high-grade gliomas (HGGs) [7,11].

Under the recently updated WHO classification system for central nervous system tumors [12], HGGs can be further differentiated by molecular markers, specifically mutations in the isocitrate dehydrogenase (IDH) gene. IDH-1 mutant astrocytomas and oligodendrogliomas tend to be less aggressive than IDH-1 wildtype glioblastomas (GBs); however, IDH-1 mutant astrocytomas can still be classified as grade IV [13]. 

HGG tumors carry incredibly high mortality rates, with only 44.4% of non-GB astrocytoma patients surviving 5 years following diagnosis [3]. This survival rate drops to 5.6% when considering only patients diagnosed with the more aggressive GB [10]. In the United States alone, thousands of people die from HGG complications each year [3]. While the last decade has seen improvements in diagnosis and clinical prognostic techniques, meaningful improvement in the mortality rate of HGGs remains elusive [11,14].

Retrospective studies have been performed to identify potential determinants of better HGG outcomes [15,16]. Considering patient demographic factors known to influence healthcare delivery and glioma outcomes [17,18], an interesting trend emerged across these studies. Patients of Hispanic ethnicity exhibited a 30% reduced incidence of gliomas compared to non-Hispanic patients [3,19,20]. Nonetheless, Hispanic patients were found to be diagnosed with HGGs 3–9 years earlier [3,19]. Despite this age disparity, HGG prognosis appeared to be improved in Hispanic patients, who demonstrated a 5-year survival rate that was up to 50% higher and a longer median time-to-death by several months [3,19,21]. 

Therefore, Hispanic ethnicity appears to have a complex association with HGG outcomes. While the longer intervals from diagnosis to death do not overcome the significant disparity in age at diagnosis, this slower progression does represent a post-diagnosis advantage in Hispanic patients. In the present study, we sought to address this burning question in glioma research: why might HGGs progress more slowly in Hispanic patients?

Many groups have highlighted that this effect has potential environmental or biologic underpinnings [1,3,21,22]. Regarding environmental factors, increased social vulnerability is known to negatively impact health outcomes across diseases [23]. Social vulnerability refers to reduced receipt of societal resources or services, which can worsen accessibility and efficacy of healthcare and lead to poorer health outcomes. For example, when a patient has poor access to safe and reliable transportation, they have reduced ability to travel to doctor’s visits and receive optimal continuing care. As prior studies have shown that oncologic outcomes worsen with increasing social vulnerability [24], we wondered if the previously observed benefit of Hispanic ethnicity to post-diagnosis disease course would still be present in a disadvantaged population. 

The goal of the present study was to lend insight to the existing discussion of whether environmental or biologic factors explain the improved HGG progression associated with Hispanic ethnicity. We accomplished this aim by retrospectively assessing the HGG-related clinical outcomes of socially vulnerable Hispanic patients compared to less vulnerable non-Hispanic patients. Through this unique approach, we aimed to determine if Hispanic patients, despite detrimental environmental disadvantages, continue to experience better post-diagnosis progression in HGGs. Our subsequent findings corroborated prior evidence of ethnicity’s impact on the clinical course of HGGs; however, for the first time, these Hispanic advantages were found to persist despite environmental disadvantages, emphasizing the need to understand the potential biology of this ethnicity-based effect.

## 2. Materials and Methods 

### 2.1. Retrospective Chart Review

Candidate patients for this retrospective study were HGG patients diagnosed and treated at a single academic medical center from 2015 through 2020. These efforts are part of an ongoing multi-institution collaboration aimed at better characterizing factors that influence HGG outcomes. The data from 72 patients were collected. For the analyses in this study, these patients were further filtered by a series of inclusion criteria: included patients were at least 18 years of age at diagnosis, reported an ethnic identity, and had a pathology-confirmed HGG (WHO III or IV). In those patients who did not receive biopsy or resection due to clinical judgment (n = 6), compelling imaging findings served to substantiate likely HGG. 

Seventeen patients were removed following inclusion criteria application. The data from the remaining 55 patients were categorized into non-Hispanic or Hispanic groups. The data from patients who were lost to follow-up were only included in those analyses which assess outcomes pertinent to dates prior to loss. For example, a patient who was lost prior to beginning chemotherapy is not included in the sample assessed for the percentage of each group receiving chemotherapy.

### 2.2. Social Vulnerability Index (SVI)

To verify socioeconomic disadvantages in the study sample, CDC/ATSDR Social Vulnerability Index (SVI) [25] scores were collected for each patient based upon home address, as previously described [24,26]. These scores utilize 2020 census data to derive a series of metrics for the local county in which the home address is located. The overall SVI score is a composite of these metrics. The individual metrics, or sub-scores, include socioeconomic status, members-of-household characteristics, racial and ethnic minority status in the local population, and housing type and transportation accessibility. The scores for each sub-score and the overall score are reported as a percentile relative to all counties in the United States. Higher percentiles represent a more severe disadvantage. 

### 2.3. Clinical Parameters and Outcomes

Baseline functional status at presentation was determined using the Eastern Cooperative Oncology Group (ECOG) performance status scale [27]. For those patients who did not receive baseline ECOG testing, researchers who were blinded to the group attribution ascertained an ECOG score using the documented history and physical evidence at presentation.

During biopsy or resection, tumor samples were collected and subsequently assessed for IDH-1 mutation status and MGMT (methylguanine-DNA-methyltransferase) gene methylation status by a validated reference laboratory. Additionally, aberrant p53 expression reflects neoplastic change, and p53 alteration is associated with more aggressive gliomas [7,12]. While p53 testing is not standard at our institution, expression status was available for 13 of the 27 recurrent IDH-1 mutant tumors. 

Time intervals, including time from diagnosis to recurrence or death, were calculated using the dates of imaging studies which identified the presence of tumor or tumor changes or available death certificates. Recurrence date was defined as the date on which imaging studies detected either progression of the treated tumor or emergence of a new tumor nodule. 

### 2.4. Statistical Analyses

The Jaque–Barre test was used to assess the normality of each dataset. As multiple outcome variables were non-normally distributed, non-parametric Mann–Whitney U tests were used to perform univariate comparisons between ethnic groups. The Fisher exact test was used to determine ethnicity-related differences in categorical variables. A multivariate analysis was performed using multiple linear regression. The SVI score was not included in the regression model due to potential collinearity of the ethnicity sub-score with the ethnicity variable that could hinder regression analysis. GraphPad PRISM 10 and Microsoft Excel (Office 365) software were used to analyze all data and produce all figures.

## 3. Results

### 3.1. Patient Cohort Demographics

Of the 72 patients treated for suspected HGGs (Table 1, “Whole Cohort”), 55 patients met the inclusion criteria (i.e., adult, provided ethnicity, and confirmed grade III or IV). A total of 33 patients identified as non-Hispanic (Table 1, “non-Hispanic”), with 13 patients identifying as being of white race (39.4%), 13 of Black race (39.4%), 3 of Asian race (9.1%), and 4 reporting other or unavailable race (12.1%). Within the non-Hispanic group, 11 patients were female (33.3%), the average BMI was 42.2, and the average age at HGG diagnosis was 65.5 years. Twenty-two patients self-identified as being of Hispanic ethnicity (Table 1, “Hispanic”). Among this group, nine patients were female (40.9%), the average BMI was 46.6, and the average age at HGG diagnosis was 57.6 years. Notably, all patients in the Hispanic group identified as being of “other” race, possibly due to their primary racial identity being their Hispanic ethnicity. No ethnicity-related differences were observed in sex composition, BMI, or the rate of treatment with chemotherapy, radiation therapy, Stupp protocol, initial biopsy, or resection following diagnosis. 

### 3.2. HGG Patients of Hispanic Ethnicity Had Higher Social Vulnerability

**Figure 1:**
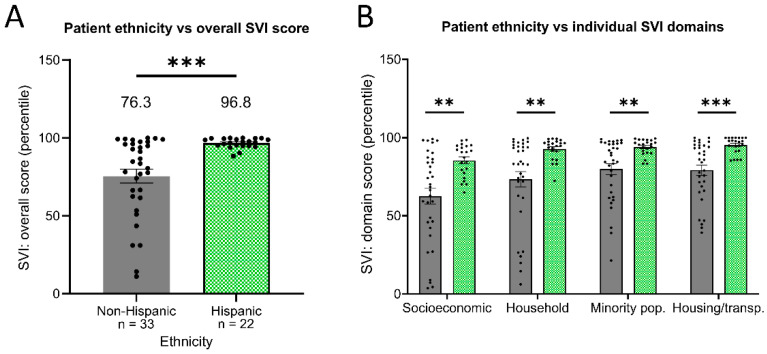
Hispanic patients exhibit higher social vulnerability than non-Hispanic patients. Generated by the CDC using the 2020 US census, social vulnerability index (SVI) scores reflect susceptibility to economic and social disadvantage. Based on home address, scores were collected for each patient and stratified by ethnicity. (**A**) The overall score represents a composite of the four domain sub-scores (**B**), which include socioeconomic status, members-of-household characteristics, racial and ethnic minority status in the local population, and housing type and transportation accessibility. Sample sizes and bar colors (Hispanic, green) in panel B mirror those of panel A. ** *p* < 0.01; *** *p* < 0.001. Error bars: standard error mean.

The average overall SVI scores for both non-Hispanic and Hispanic patients were in the upper quartile (>75th percentile), meaning that the average patient in both groups resided in one of the top 25% most socially disadvantaged counties in the US. However, Hispanic patients had significantly higher overall SVI scores than non-Hispanic patients (Figure 1A; Hispanic: 96.8 + 0.7; non-Hispanic: 76.3 + 4.6; *** *p* = 0.0002). Each of the SVI sub-scores (Figure 1B) demonstrated similar disadvantages, including socioeconomic status (Hispanic: 85.4 + 0.02; non-Hispanic: 62.6 + 0.05; ** *p* = 0.003), members-of-household characteristics (Hispanic: 92.8 + 0.01; non-Hispanic: 73.3 + 0.05; ** *p* = 0.003), minority proportion of local population (Hispanic: 93.9 + 0.01; non-Hispanic: 79.8 + 0.04; ** *p* = 0.006), and housing type and transportation accessibility (Hispanic: 95.2 + 0.01; non-Hispanic: 79.2 + 0.03; *** *p* = 0.0001). 

### 3.3. Clinical Outcomes of HGGs Differed in Patients of Hispanic Ethnicity

**Figure 2:**
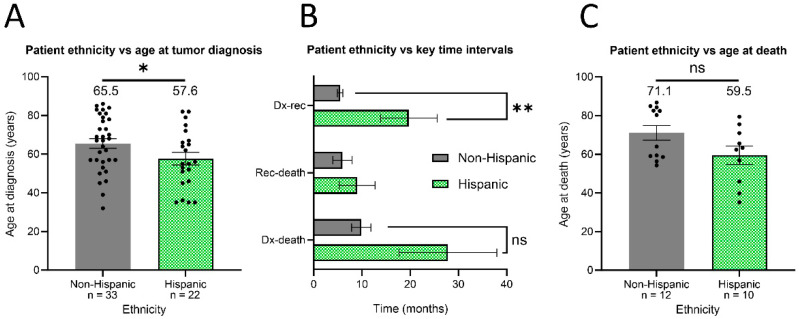
Hispanic patients with high-grade gliomas are younger at diagnosis and have longer intervals before first recurrence. Patients with high-grade gliomas (WHO III or IV) were assessed for incidence and outcome characteristics. (**A**) The average age at diagnosis, defined as each patient’s age on the date of first positive imaging study, was found for non-Hispanic and Hispanic patients. (**B**) For both non-Hispanic and Hispanic patients, the average time interval was calculated between multiple dates, including the date of diagnosis (Dx), the date of first recurrence or progression (rec: defined as suspicious tumor re-emergence or growth on post-treatment imaging study), and the date of death for those patients with available death certificates. (**C**) The average age at death was collected for non-Hispanic and Hispanic patients. * *p* < 0.05; ** *p* < 0.01. Error bars: standard error mean.

The average age at which Hispanic patients were diagnosed with HGGs was roughly eight years younger than that of non-Hispanic patients (Figure 2A; Hispanic: 57.6 + 3.2; non-Hispanic: 65.5 + 2.5; * *p* < 0.05). Hispanic patients also exhibited a significantly longer interval between diagnosis and first HGG recurrence (Figure 2B; Hispanic: 19.7 + 5.9 months (n = 13); non-Hispanic: 5.5 + 0.6 months (n = 19); ** *p* = 0.001). Again, this finding occurred without observed differences in which treatments patients received (Table 1).

This 14-month advantage in time to recurrence, however, did not correspond to an altered interval from recurrence to death (Figure 2B; Hispanic: 9.0 + 3.7 months (n = 5); non-Hispanic: 6.0 + 2.0 months (n = 10); *p* = 0.513). Similarly, no statistically significant difference was observed in the interval from diagnosis to death, though the average time to death appeared to be 18 months longer in Hispanic patients (Figure 2B; Hispanic: 27.8 + 10.1 months (n = 10); non-Hispanic: 9.8 + 2.0 months (n = 13); *p* = 0.376). Among those patients with available death certificates, no ethnicity-based difference was found in the age at death, though Hispanic patients did appear to be at least ten years younger on average (Figure 2C; Hispanic: 59.5 + 4.7 years (n = 10); non-Hispanic: 71.1 + 3.8 years (n = 12); *p* = 0.123).

### 3.4. Hispanic Ethnicity Continued to Predict a Longer Time to Recurrence among More Aggressive IDH-1 WT GBs

**Figure 3:**
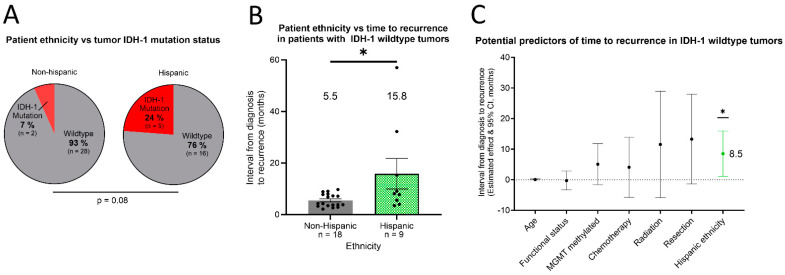
Standard clinical and biologic prognostic factors do not explain the slower progression of tumors in Hispanic patients. Among high-grade gliomas, IDH-1 mutant tumors have a more favorable prognosis. (**A**) The rates of IDH-1 mutant tumors in the non-Hispanic and Hispanic patient groups were calculated. Only the data from patients in whom tumor IDH-1 testing was performed were included. (**B**) As IDH-1 wildtype tumors have a poorer prognosis, the assessment of ethnicity-related differences in the average time from diagnosis to recurrence or progression was repeated. Error bars reflect the standard error mean. (**C**) Among the patients with IDH-1 wildtype tumors, a multiple linear regression analysis was performed to identify independent predictors of the diagnosis–recurrence interval. The candidate predictors included in the model were age at diagnosis, baseline functional status (assessed by ECOG scale), MGMT methylation of the tumor, receipt of each primary treatment modality, and patient ethnicity. Error bars in (**C**) represent the bounds of the 95% confidence interval around the estimated effect of each predictor on interval length. * *p* < 0.05.

Of the 55 patients in this study, IDH-1 mutation testing results were available for all patients except 4 (3 non-Hispanic and 1 Hispanic). Among the Hispanic patients, five (23.6%) had tumors which were IDH-1 mutation-positive (Figure 3A). Comparatively, only two (6.7%) non-Hispanic patients had tumors which were IDH-1 mutation-positive (Figure 3A). While these frequencies did not significantly differ (*p* = 0.08), we sub-set the data from those patients with aggressive IDH-1 wildtype tumors to further investigate if an increased prevalence of less-aggressive IDH-1 mutated tumors might explain the observed time-to-recurrence advantage in Hispanic patients. 

Further supporting their aggressive nature, aberrant expression of p53, a key tumor suppressor [7], was found in each IDH-1 wildtype tumor in which p53 status was assessed on post-op pathology. The time from diagnosis to recurrence remained 10 months longer in Hispanic patients with IDH-1 wildtype tumors compared to their non-Hispanic counterparts (Figure 3B; Hispanic: 15.8 + 5.9 months (n = 9); non-Hispanic: 5.5 + 0.6 months (n = 18); * *p* = 0.034). 

A multivariate analysis was then performed to determine the predictive ability of key prognostic variables on time to recurrence (Figure 3C). The multiple linear regression model incorporated several candidate predictors (“estimated effect on months until recurrence”, “[95% confidence interval]”), including age at diagnosis (0.1, [−0.1, 0.3]), ECOG functional status at presentation (−0.3, [−3.3, 2.8]), tumor MGMT methylation positivity (5.1, [−1.6, 11.7]), chemotherapy treatment (4.1, [−5.6, 14.0]), radiation treatment (11.5, [−5.8, 28.9]), and resection treatment (13.3, [−1.4, 27.9]). However, only Hispanic ethnicity independently predicted time from diagnosis to recurrence (8.5, [1.1, 15.9]; * *p* = 0.027). Therefore, independent of other established prognostic factors, the model predicts that Hispanic patients experience an additional eight months until recurrence of IDH-1 wildtype HGG tumors.

## 4. Discussion

Across multiple large-scale studies, Hispanic patients diagnosed with HGGs exhibit improvements in some prognostic intervals compared to non-Hispanic patients [1,3,19,22]. We sought to better understand why this effect occurs. Our findings replicated known differences in the age at diagnosis and post-diagnosis outcomes of HGGs in Hispanic patients; however, for the first time, this effect was demonstrated in a socially vulnerable sample of Hispanic patients. As our academic medical center is located in a region with primarily disadvantaged patients, performing the retrospective analysis of HGG patients treated at our facility provided ideal access to the study population of interest. 

Using the SVI [26], we confirmed that Hispanic patients in this study’s cohort were socially vulnerable. In fact, these patients lived within the top 5% most disadvantaged communities in the United States. Additionally, the Hispanic patients exhibited higher social vulnerability than the non-Hispanic patients across all SVI sub-scores. These findings established that this study’s patient sample would facilitate our subsequent analysis which sought to answer whether disadvantaged Hispanic patients see better HGG disease course following diagnosis.

In our study, socially vulnerable Hispanic patients were found to be diagnosed with HGG 8 years earlier than less vulnerable non-Hispanic patients; this finding corroborated previous studies that found Hispanic patients, without considering their vulnerability, were diagnosed 3–9 years earlier than non-Hispanic patients [3,19]. However, we did not detect the previously established increased length from diagnosis to death in this Hispanic patient sample. One potential limitation of our study was the sample size reduction induced by our strict use of data only from those patients with available death certificates. 

To ensure date-of-death accuracy within the confines of limited data availability, we likely reduced the power of our analyses by not inferring dates of death. Such a limitation is likely why the average time to death appeared much longer in Hispanic patients but was not statistically significant: the variability was too great in the resultingly reduced sample size. Similarly, those non-Hispanic patients with available death certificates belonged to the portion of this group that was older at diagnosis. This skewedness meant that the average age at death was likely erroneously inflated, explaining the apparent 5-year difference between the average ages of diagnosis and death in non-Hispanic patients. On the other hand, the difference of 2 years between the average age at diagnosis and death in the Hispanic group roughly approximated the observed interval of 28 months between diagnosis and death in this group. 

Given its single-institution design and strict inclusion criteria, the present study featured a sample size which likely limited its statistical power. However, these exploratory analyses were the necessary first steps toward understanding the role of environmental factors in the effects of ethnicity on HGG outcomes. In order to strengthen the statistical power of our analyses, future multi-institution studies will be needed. Furthermore, a multi-institutional study would enable the comparison of Hispanic patients from vulnerable and non-vulnerable backgrounds, thereby building on our findings by optimally controlling for ethnicity while assessing the impacts of social vulnerability. 

Again, highlighting the ultimate age disparity in HGG survival outcomes experienced by Hispanic patients, the average Hispanic patient in this study died at an age (59.5 years) which was younger than the average age (65.5 years) at which non-Hispanic patients were diagnosed. While Hispanic patients may live longer post-diagnosis, the significantly earlier age of diagnosis means that they die from HGG at a much younger age. Future work must seek to understand why Hispanic patients are diagnosed at a significantly younger age, as evidenced by prior research and the present study. Nonetheless, longer HGG survival intervals that are associated with ethnicity offer an opportunity to better understand the factors which influence HGG progression.

While previous large-scale studies established that Hispanic patients experienced longer survival following the diagnosis of HGGs, a strength of the present study’s single-institution design was the access to longitudinal observation outcomes which are unavailable in national databases. As a result, this study was the first to demonstrate an impact of Hispanic ethnicity on the interval from diagnosis to recurrence. In the context of post-diagnosis outcomes, HGG progression potentially occurs slower in Hispanic patients, and this difference could explain previously observed survival benefits.

As no prior study has reported such ethnicity-related changes in tumor natural history, we employed an inclusive definition of recurrence. Recurrence represented either the progression of the initial tumor or the emergence of a new tumor nodule. Both events required radiologist confirmation on follow-up imaging studies. This approach provided an ample sample size to enable our univariate and multivariate comparisons of time to recurrence.

The present study successfully detected increased time to HGG recurrence in a Hispanic community with confirmed social vulnerability for the first time. As IDH-1 mutation status distinguishes more aggressive GBs from other HGGs, we wondered if the time-to-recurrence increase in Hispanic patients could be explained by increased IDH-1 mutated (non-GB) tumors in this group. Though a higher proportion of Hispanic patients had IDH-1 mutated tumors compared to non-Hispanic patients, this difference was not statistically significant. This frequency comparison could have been constrained by sample size limitations, so we resolved to repeat the time-to-recurrence analysis in only those patients with more aggressive IDH-1 wildtype tumors. Frequent p53 aberrations were also found as further confirmation of the aggressive nature of these tumors.

Hispanic patients with GBs yet again demonstrated an average interval from diagnosis to first recurrence that was longer than that of non-Hispanic patients by 10 months. Furthermore, Hispanic ethnicity was found to predict time to recurrence independent of established clinical prognostic factors, including age at diagnosis, baseline functional status [15], tumor MGMT methylation [7], and treatments received. Therefore, not only does the time-to-recurrence advantage occur in Hispanic patients despite severe social vulnerability, but it also occurs independent of other prognostic factors in the most aggressive form of HGGs.

Taken together, the present study applied a unique design to detect, for the first time, that the post-diagnosis advantages seen in Hispanic patients persist despite environmental burdens. Importantly, our findings do not imply that Hispanic ethnicity is associated with overall better HGG prognosis. Hispanic patients likely die at a younger age, because the recurrence advantages of several months are not equal in magnitude to the age-at-diagnosis disadvantage of several years. This disparity complicates the influence of ethnicity on HGG outcomes. On the one hand, Hispanic patient outcomes are worse because they are affected by HGGs at a younger age; however, Hispanic patients experience more recurrence-free months. The age disparity undoubtedly takes years of life away from Hispanic patients and their families, and it warrants thorough investigation in future studies. 

However, the slower tumor progression potentially gives Hispanic patients more time with an improved quality of life following diagnosis. Moreover, this slower progression to recurrence occurs independent of social vulnerability. These findings point to a diminished role of environmental factors and places emphasis on the need for further basic science investigations of factors predisposing Hispanic patients to slower HGG progression. Such areas of investigation include genetic studies ascertaining oncogenic risk variants [28,29] or cellular variations which alter the tumor microenvironment to delay the rate at which glial tumors advance [30]. Uncovering the potential biologic mechanisms of Hispanic ethnicity’s apparently protective effect on time to glioma recurrence could revolutionize care for all patients with HGGs.

## 5. Conclusions

HGGs are among the most devastating oncologic pathologies, and increasing focus is being placed on understanding the factors which influence patient outcomes. Notably, Hispanic ethnicity is known to be associated with improved HGG clinical progression, an effect potentially mediated by environmental or biologic factors. In this study, we sought to determine if this post-diagnosis advantage occurred in Hispanic patients despite severe social vulnerability, an environmental determinant of healthcare outcomes.

We found that Hispanic patients from highly disadvantaged communities still exhibited an improved post-diagnosis outcome compared to non-Hispanic patients, as demonstrated by a 14-month longer interval from diagnosis to recurrence. Even when considering only patients with the more aggressive GB sub-type of HGGs, socially vulnerable Hispanic patients saw a delay in recurrence, and Hispanic ethnicity was found to predict time to GB recurrence independent of established prognostic considerations.

These novel findings build upon the existing literature by establishing that environmental factors which typically predispose patients to poor outcomes do not obscure the longer time to recurrence seen in Hispanic patients. Our findings emphasize the need for future basic research studies ascertaining the potential biological, rather than environmental, etiology of Hispanic ethnicity’s strong influence on post-diagnosis outcomes in HGGs.

## Figures and Tables

**Table 1:** Patient demographics and treatment summary. (*) statistical significance of *p* < 0.05.

		Whole Cohort		Non-Hispanic		Hispanic		*p*-Value
		Mean	SEM	Mean	SEM	Mean	SEM	
Number		72	-	33	-	22	-	-
Sex (% female)		43.1	-	33.3	-	40.9	-	0.6252
Age at diagnosis		59.5	2.2	65.5	2.5	57.6	3.2	0.0499 *
Body mass index		40.5	1.6	42.2	2.3	46.6	2.8	0.1776
IDH1 mutation positive (%)		15.9	-	6.7	-	23.8	-	0.0800
Social vulnerability index	Overall (percentile)	85.6	2.5	76.3	4.6	96.8	0.7	0.0002 *
	“highly vulnerable” (% of sample)	93.1	-	87.9	-	100	-	0.1414
Race (%)	White	20.8	-	39.4	-	0.0	-	-
	Black	23.6	-	39.4	-	0.0	-	-
	Asian	4.2	-	9.1	-	0.0	-	-
	Other	45.8	-	9.1	-	100.0	-	-
	Unavailable	5.6	-	3.0	-	0.0	-	-
Ethnicity (%)	Hispanic	40.3	-	0.0	-	100.0	-	-
	Non-Hispanic	51.4	-	100.0	-	0.0	-	-
	Unavailable	8.3	-	0.0	-	0.0	-	-
Treatment (%)	Chemotherapy	63.8	-	76.2	-	71.0	-	0.6770
	Radiation therapy	81.5	-	85.7	-	89.7	-	0.6861
	Stupp protocol	67.3	-	88.2	-	60.0	-	0.0809
	Biopsy	14.1	-	18.2	-	3.1	-	0.1460
	Resection	77.5	-	77.3	-	84.4	-	0.7230

## Data Availability

The data are available upon reasonable request from the authors.
